# *Onosma* L. as a source of anticancer agents: phytochemistry to mechanistic insight

**DOI:** 10.37349/etat.2022.00109

**Published:** 2022-10-31

**Authors:** Ajay Kumar, Shivani Attri, Sandeep Kaur, Hardeep Singh Tuli, Reena V. Saini, Adesh Kumar Saini, Manoj Kumar, Satwinderjeet Kaur

**Affiliations:** 1 Department of Botanical and Environmental Sciences, Guru Nanak Dev University, Amritsar 143001, India; 2 Department of Biotechnology, Maharishi Markandeshwar (Deemed to be University), Mullana 133207, India; 3 Department of Chemistry, Maharishi Markandeshwar University, Sadopur-Ambala 221304, India; NGO Praeventio, Estonia

**Keywords:** Alkaloids, anti-cancer, anti-inflammatory, Boraginaceae ethnopharmacological, naphthoquinones, shikonin

## Abstract

*Onosma* (*O*.) is a genus of perennial flowering plants in the family Boraginaceae with approximately 250 species widely dispersed in temperate, tropical, and subtropical areas. It is traditionally used to treat rheumatism, fever, asthma, stomach irritation, and inflammatory ailments. The bioactive constituents present in the genus *O*. include benzoquinones, naphthazarins, alkaloids, phenolic, naphthoquinones, and flavonoids whereas shikonins and onosmins are the most significant. The review compiled contemporary research on *O*. L., including its distribution, morphology, traditional applications, phytochemistry, ethnopharmacology, and toxicology. This review also highlights a few critical challenges and possible future directions for *O*. L. research. Modern research has demonstrated a wide range of pharmacological effects of different species of *O*. L., including anti-diabetic, anticancer, anti-inflammatory, and cardiovascular protective. However, the studies on the *O*. genus are still not fully explored, therefore, researchers need to discover novel products with their toxicity studies, molecular mechanism, and associated side effects. Future exploration of potent constituents from this genus and clinical trials are required to explore its pharmacological importance.

## Introduction

*Onosma* (*O*.) L. (Boraginaceae) is now one of the biggest genera in the tribe Lithospermeae Dumort. More than 230 species of Boraginaceae exist throughout Asia and Europe [1–4]. There are numerous species of *O*. present throughout the globe such as 105 species in Turkey [5], 54 species in Iran [6], 8 species in Pakistan [7], more than 15 species in India [8], 33 and 34 species in Russia and adjacent states [9]. There are now 250 species in the genus *O*. as a result of several studies that have been directed in recent years [10–14]. Linnaeus established the botanical name “*onosma*” for this genus, a translation from the Latin word “osma” which derives from the Greek word “osma”, meaning “smell”, which is regarded to be feminine [15]. All species are commonly referred to as “rock garden plants” and grow in sunny, dry, or damp settings, typically in cracks on rocks [10]. *O*. L. is a genus with many species and a complex network of morphological and karyological diversity with very contentious taxonomic treatments within groups. Numerous identical taxa were defined based on slight physical changes, which frequently led to confusion. Additionally, their distribution in the European region is rather fragmented, and classifications have frequently been made using limited studies of geographical distribution, which appears partially artificial. Further, research is necessary to observe whether new data will be helpful as references in a future classification. There is a total of 387 scientific nomenclature of species scores for this genus listed in “The Plant List” of the Royal Botanic Garden, Kew, and Missouri Botanical Garden, and out of them only 37 species names are approved and additional 19 plants scientific names are infra-specific rank i.e., any taxon below the rank of species [16]. These results show that just 9.6% of names are accepted, 6.2% are synonyms, and 84.2% of names/titles are indeed awaiting evaluation. The species of *O*. are widely used in traditional medicine to treat rheumatism, bladder pain, kidney irritation, heart palpitations, laxatives, blood disorders, bronchitis, leucoderma, fever, wounds, burns, piles, urinary calculi, etc. [17, 18]. The flowers of the plant *O*. *hispidum* Wall. is used as a heart tonic and stimulant, whereas roots are used to overcome fever, pain alleviation, bites, wounds, infectious disorders, and stings. In addition, it is also used for topical application to treat cutaneous ailments [19]. *O*. *bracteatum* Wall. also called Gaozaban in the Unani medical system and sedge in the Middle East, has historically been consumed as a tonic to increase the body’s immune system while controlling urine production [20]. It has also been taken as a tonic, alterative, diuretic, spasmolytic, and demulcent, to treat asthma and bronchitis [21]. In addition, a decoction is consumed in folk medicine to cure wounds, skin conditions as well as leprosy, syphilis, restlessness in febrile excitement, rheumatism, soothing excessive thirst, irritated bladder, palpitations of the heart, stomach, and strangury [22]. The leaf powder of the plant *O*. *echioides* DC. is alternatively given to children as a purgative. Flowers are consumed as a cordial stimulant for curing rheumatism and heart palpitations whereas roots are used to treat infectious skin problems [23, 24]. *O*. species show all activities due to the presence of phytochemicals such as shikonin, pyrrolizidine alkaloids, phenolic, naphthoquinones, and flavonoids [8, 19]. Scientific studies have supported some of the traditional applications of diverse species and demonstrated their potential as antioxidants [25], anti-inflammatory agents [26], spasmolytic agents, antibacterial agents [27–29], analgesic agents, and wound healers [30, 31].

## Geographical distribution of *O*. L.

*O*. L. species are primarily found in Asia, the Mediterranean region, Eurasia, and Europe, particularly in China, Iran, Syria, India, Turkey, Pakistan, and Sri Lanka [31–34]. Kumar et al. [35] highlighted how distribution patterns of different *O*. L. species vary geographically. An indigenous species of *O*. *pyramidale* Hook. and *O*. *bracteatum* is found in India’s Kali Valley, Eastern Kumaun (Uttarakhand), and the region of Kashmir and Himalayas [36–38]. Anatolia is a significant center of origin for *O*. L. with 101 species (107 taxa), 50 of which are endemic, including one variety from Turkey [39]. *O*. *tornensis*, whose number of localities is stable and *O*. *visianii* are the most common species in Slovakia, whereas *O*. *arenaria* and *O*. *pseudoarenaria* are listed as endangered species in Slovakia due to climate reasons [40]. *O*. *visianii* Clem flourishes in Romania’s desolate regions of Dobrogea, steppe, and calcareous soils while *O*. *setosu*, *O*. *arenaria*, *O*. *pseudoarenaria*, and *O*. *viride* are endemic, spreading in barren areas [41–43]. In addition, the concentration and occurrence of phytoconstituents in different species of *O*. also vary according to geographical distribution. The presence of phytochemicals depends upon the origin of biomass, geographical site, availability of light, and other climatic factors [44].

## Morphology

*O*. L., a genus consists of biennial/perennial herbs, rough and petiolate leaves with entire edges [39, 45, 46]. Flowers have actinomorphic, pedicellate, or sessile and branches make a panicle, which is often elongated in fruit and bracteate. Cymes are scorpioid and solitary at the stem apex [47]. The calyx is divided into five equal lobes that are linear or linear-lanceolate, frequently increased during anthesis, and extending to or close to the root. Corolla is tubular, gradually extended from the base upward and unappendaged with white, blue, yellow, or red color [48], retrorse shape [49]. In the family Boraginaceae, the number of genera has enlarged base setae and some *O*. species have setae with 4–20 rays rising from the base called stellate setae [50]. The hairs occasionally lack central seta, but when they do, it is typically seen as longer and stouter in comparison to rays [51]. Existence or absenteeism of the stellate setae is extensively considered a key feature of this genus, but an extensive discrepancy in the presence, frequency, and length of stellate setae was observed in numerous species. The genus provides significant taxonomic challenges that cannot be resolved without experimental research, especially in central and south-eastern Europe.

## Phytochemistry

**Figure 1.**
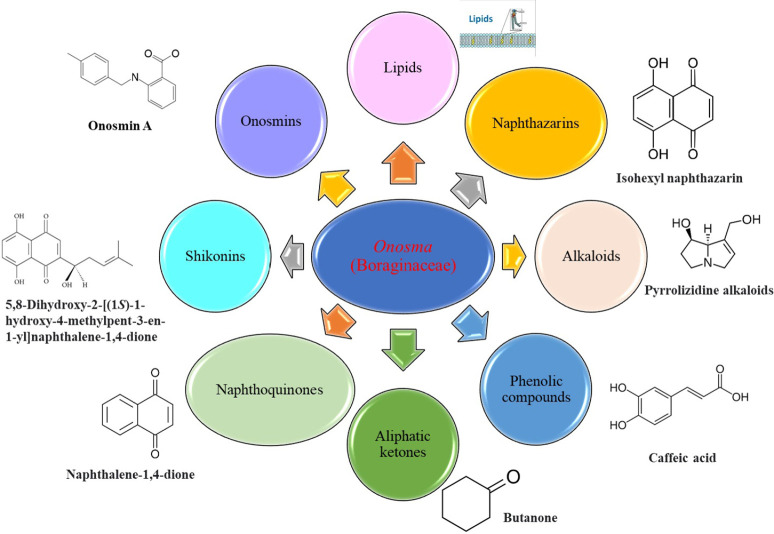
Different phytoconstituents present in the genus *O*.

The literature review found that the genus *O*. L. has received relatively little attention in phytochemistry, with only a few reports of phenolic compounds, alkaloids, and naphthoquinones (Table 1) [33]. The plants of *O*. genus, have several categories of secondary metabolites like alkaloids, naphthazarins, aliphatic ketones, flavones lipids, phenolic compounds, and naphthoquinones (Figure 1) [34]. The naturally occurring isohexenylnaphthazarins alkannins and shikonins are chiral constituents primarily distributed in the outer layer of roots among several taxa belonging to the Boraginaceae family [51]. El-shazly et al. [3] reported that alkaloids isolated from *O*. *arenaria* are structure elucidation by mass spectroscopy and ^1^H- and ^13^C-nuclear magnetic resonance (NMR) analysis and characterized as uplandicine, 1,2-unsaturated pyrrolizidine alkaloids. Ahmad et al. [52] reported Hispidone, a novel flavanone procured from the species *O*. *hispida* for the first time and its structure was assigned as (2*S*)-5,2’-dihydroxy-7,4’,5’-trimethoxyflavanone. Moreover, (2*S*)-5,2’-dihydroxy-7,5’-dimethoxyflavanone, 4-hydroxy benzoic acid, and benzoic acid were discovered first from this species. Mehrabian et al. [47] and Özgen et al. [53] procured deoxyshikonin, 3 hydroxy isovaleryl shikonin, acetyl shikonin, and 5,8-*O*-dimethyl acetyl shikonin from the extract of *O*. *argentatum* using a solvent ration of n-hexane-dichloromethane (1:1). Further, these isolated compounds subjected to ^1^H- and ^13^C-NMR and mass spectroscopy analysis. Naz et al. [54] proposed that *O*. *hispidum* ethanolic extract consists of 4-hydroxy-3-methoxy cinnamic acid (ferulic acid) and 4-hydroxy-3-methoxy benzoic acid (vanillic acid) and it is characterized using ^1^H-NMR, ^13^C-NMR, and high-performance liquid chromatography (HPLC) techniques. The compounds Onosmins A and B are derived from the species *O*. *hispidum* Wall. Their structures and spectroscopic research include two-dimensional (2D) NMR [55]. Sagratini et al. [56] reported the alkannin or shikonin in *O*. *echioides* with some derivatives of naphthoquinone such as arnebin-6 and 5,8-dihydroxy-2-(4-methyl-6-oxo-5,6-dihydro-2H-pyran-2-yl) naphthoquinone with structure elucidation using HPLC-mass spectrometry (MS). The diethyl ether extract of plants *O*. *bracteosum* and *O*. *thracicum* showed the presence of oleic and α-linolenic acids but their maximum amount was found in the native *O*. *bracteosum* whereas higher quantities of other fatty acids and tocopherol observed in species *O*. *thracicum* [57]. *O*. *heterophylla* consists of a suitable amount of caffeic acid, rutin, rosmarinic acid, kaempferol, and *O*-coumaric acid in the methanol, ethyl acetate, and water extracts which are responsible for antioxidant potential [58]. Kundaković et al. [59] demonstrated that *O*. *arenaria* exhibits cytotoxicity against HeLa (human cervix adenocarcinoma cells) and K562 (leukaemia) cell lines due to the presence of phytoconstituents. Vukic et al. [60] reported that among the different compounds of the plant *O*. *visianii*, α-methylbutyryl shikonin and acetyl shikonin exhibit higher cytotoxicity towards MDA-MB-231 (human breast carcinoma) cell line. In addition, Saravanakumar et al. [61] revealed methanolic extract of *O*. *isaurica* and *O*. *bracteosa* showed more substantial free radical quenching potential because of the present compounds such as oleic acid, α-linolenic acids, alkannin, shikonin, flavonoids, ferulic and vanillic acids. A study by Saravanakumar et al. [62] revealed that methanolic extract of *O*. *lycaonica* showed good antioxidant potential as assessed by 2,2’-azino-bis(3-ethylbenzothiazoline-6-sulfonic acid) (ABTS) assay due to the presence of phytoconstituents apigenin, luteolin, ferulic acid, pinoresinol, apigenin 7-glucoside, 4-hydroxybenzoic acid, eriodictyol, rosmarinic acid, luteolin 7-glucoside, vanillin, caffeic acid and (+)-catechin 3,4-dihydroxyphenylacetic acid.

**Table 1.** Phytoconstituents procured from genus *O*.

**Serial No.**	**Compound**	**Molecular formula**	**Molecular weight**	**Structure**	**Plant used**	**References**
1	Hispidone (2*S*)-5,2′-dihydroxy-7,4′,5′-trimethoxyflavanone	C_30_H_48_O_4_	472.70 g/moL	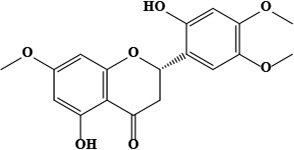	*O*. *hispidum*	[52]
2	7-Acetylretronecine	C_10_H_15_NO_3_	197.23 g/moL	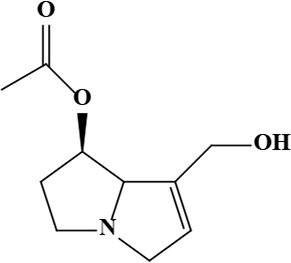	*O*. *arenaria*	[3]
3	Uplandicine [(7*R*,8*R*)-7-acetyloxy-5,6,7,8-tetrahydro-3*H*-pyrrolizin-1-yl] methyl (2*R*)-2,3-dihydroxy-2-[(1*S*)-1-hydroxyethyl]-3-methylbutanoate	C_17_H_27_NO_7_	357.40 g/moL	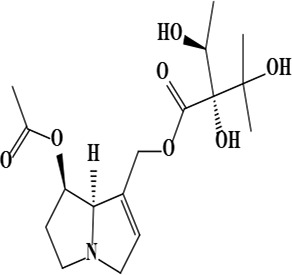	*O*. *arenaria*	[3]
4	Onosmin B (methyl 2 [(4-methyl benzyl) amino] benzoate)	C_16_H_17_NO_2_	255.31 g/moL	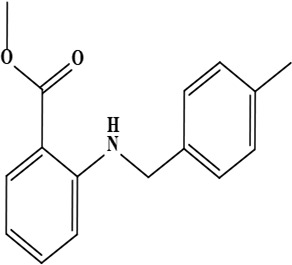	*O*. *hispidum*	[55]
5	Onosmin A (2-[(4-methylbenzyl) amino] benzoic acid)	C_15_H_15_NO_2_	241.28 g/moL	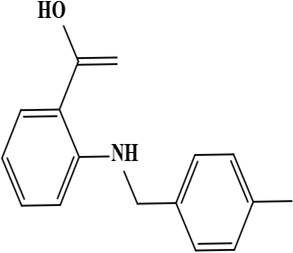	*O*. *hispidum*	[55]
6	Onosmone (9*R*)-4,9-dihydroxy-7,12,15-trimethoxytricyclo[9.4.0.0^{3,8}]pentadeca-1(15),3,5,7,11,13-hexaen-2-one	C_18_H_18_O_6_	330.33 g/moL	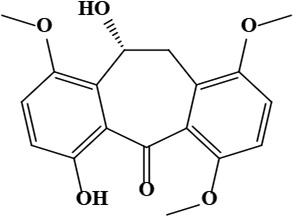	*O*. *limitaneum*	[55]
7	Bauerenone (4*aR*,6*aS*,6*bS*,8*aR*,11*R*,12*S*,12*aR*,14*aR*,14*bR*)-4,4,6*a*,6*b*,8*a*,11,12,14*b*-octamethyl-1,2,4*a*,5,7,8,9,10,11,12,12*a*,13,14,14*a-*tetradecahydropicen-3-one	C_30_H_48_O	424.70 g/moL	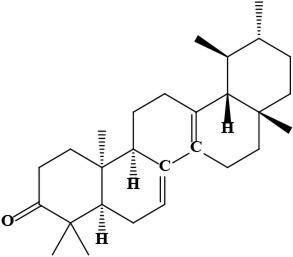	*O*. *limitaneum*	[55]
8	Ferulic acid (4-hydroxy-3-methoxy cinnamic acid)	C_10_H_10_O_4_	194.06 g/moL	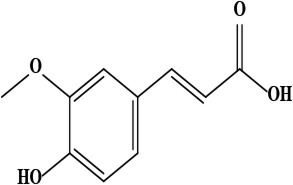	*O*. *hispidum*	[54]
9	Vanillic acid (4-hydroxy-3-methoxy benzoic acid)	C_8_H_8_O_4_	168.15 g/moL	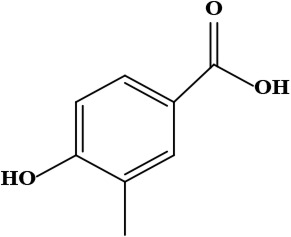	*O*. *hispidum*	[54]
10	Naphthoquinone 5,8-dihydroxy-2-(4-methyl-6-oxo-5,6-dihydro-2*H*-pyran-2-yl)-[1,4]naphthoquinone	C_16_H_12_O_6_	300.06 g/moL	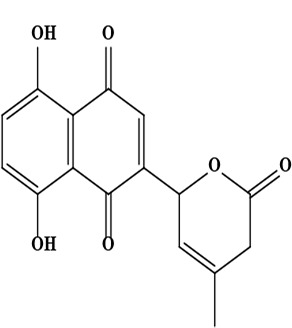	*O*. *echioides*	[56]
11	Deoxyshikonin (5,8-dihydroxy-2-(4-methylpent-3-enyl)naphthalene-1,4-dione)	C_16_H_16_O_4_	272.30 g/moL	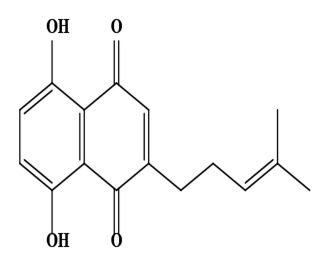	*O. echioides*	[56]
12	Acetylshikonin [1-(5,8-dihydroxy-1,4-dioxonaphthalen-2-yl)-4-methylpent-3-enyl] acetate	C_18_H_18_O_6_	330.30 g/moL	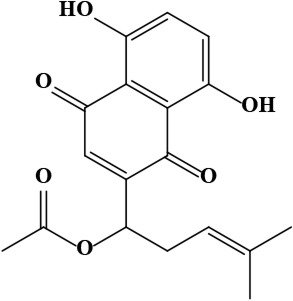	*O*. *armeniacum*	[63]
13	1,4-naphthoquinone naphthalene-1,4-dione	C_10_H_6_O_2_	158.02 g/moL	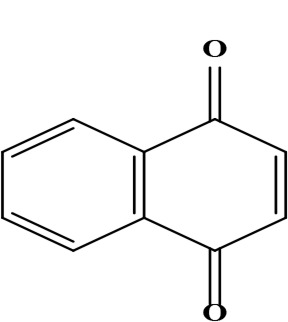	*O*. *echioides*	[64]
14	Isobutyrylshikonin 1-(5,8-dimethoxy-1,4-dioxo-1,4-dihydronaphthalen-2-yl)-4-methylpent-3-en-1-yl 2-methylbutanoate	C_23_H_28_O_6_	400.19 g/moL	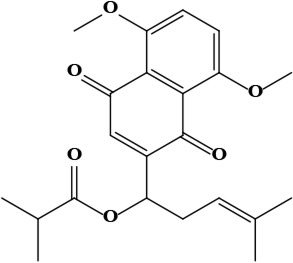	*O*. *visianii*	[65]
15	Naphthoquinone (*E*)-2-(4-hydroxy-4-methylpent-2-en-1-yl)-5,8-dimethoxynaphthalene-1,4-dione	C_18_H_20_O_5_	316.34 g/moL	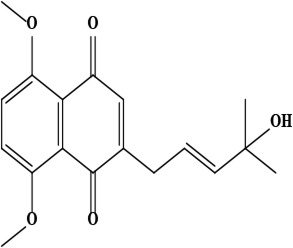	*O*. *visianii*	[65]
16	β,β-dimethylacrylalkannin	C_21_H_22_O_6_	370.40 g/moL	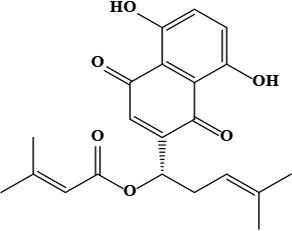	*O*. *dichroantha*	[66]
17	2,3-dihydro-3,5-dihydroxy-7-methoxyflavone	C_16_H_14_O_5_	286.08 g/moL	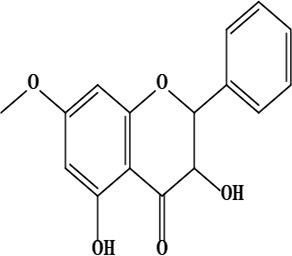	*O*. *chitralicum*	[67]
18	7,4’-dihydroxy-3’-methoxyisoflavone	C_16_H_12_O_5_	284.07 g/moL	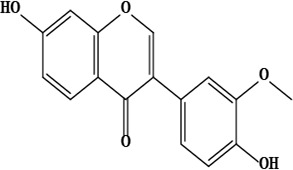	*O*. *chitralicum*	[67]
19	Ehretiquinone ((1*R*,9*R*,10*S*)-6-hydroxy-16-methyl-10-[(1*E*)-3-methylbuta-1,3-dienyl]-2-oxatetracyclo[7.5.3.0^1,10^.0^3,8^]heptadeca-3(8),4,6,12,16-pentaene-11,14-dione)	C_22_H_20_O_4_	348.14 g/moL	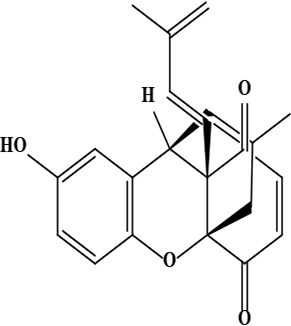	*O*. *bracteatum*	[68]

## Ethnopharmacology of genus *O*. with special reference to cancer

**Figure 2.**
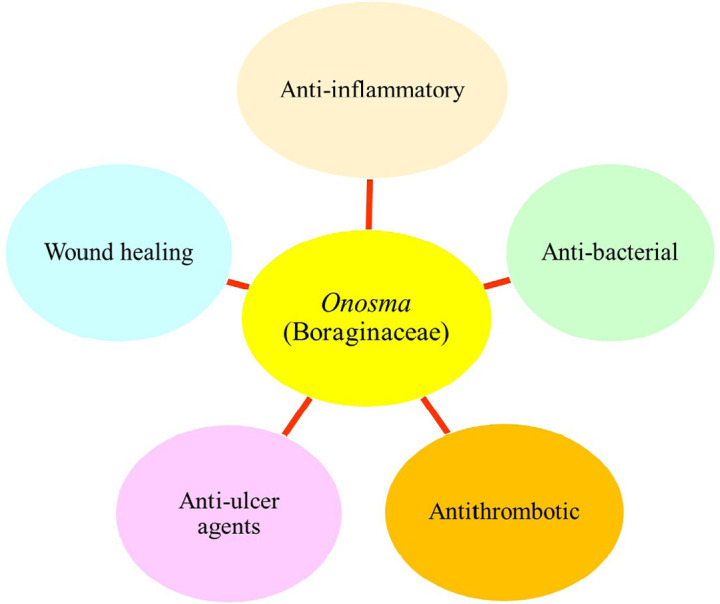
Different medicinal properties of the *O*. genus

The presence of flavonoids, vanillic acids, alkannin, shikonin, ferulic and other compounds in *O*. L. plants has many medicinal properties such as analgesic, anti-inflammatory, wound-healing, and antibacterial (Figure 2) [35]. Furthermore, according to a reported study [33, 69], numerous phytochemicals in the *O*. genus significantly reduce inflammation and ease pain without toxic effects.

Sharma et al. [70] investigated that methanolic extract of *O*. *echioides* has reduced oxidative damage induced by 7,12-dimethylbenz(a)anthracene (DMBA) in the skin of Swiss albino mice, which might be due to the presence of two key compounds (alkannins and shikonins). Numerous studies and experimental data have proved that consuming plants and their bioactive ingredients with antioxidant properties will boost the antioxidant defense system against reactive oxygen species (ROS) and minimize oxidative stress [71]. *O*. *visianii* showed growth inhibitory effects on HCT-116 (human colorectal carcinoma) and MDA-MB-231 (human breast carcinoma) cells via inducing cell cycle arrest at the G_2_ phase. This effect might be due to the presence of shikonin derivatives [72]. A study by Ahmad et al. [55] concludes that the compound Onosmin A, an aminobenzoic acid procured from *O*. *hispida*, can inhibit enzyme lipoxygenase in a dose-dependent manner. Cadirci et al. [73] found that hexane-dichloromethane root extract of *O*. *armeniacum* has a protective effect against oxidative stress generated by ethanol in the stomach of Wistar rats by inhibiting malondialdehyde (MDA) levels in stomach tissue. Toker et al. [63] conclude that acetyl shikonin collected from species *O*. *armeniacum* has an antiulcer effect by increasing antioxidant activity and level of nitric oxide and suppressing oxidative marker (malondialdehyde) in the stomach of male Wistar rats. Rinner et al. [74] reported that petroleum ether fraction of plant *O*. *paniculatum* showed antiproliferative potential via enhancing the procaspase-3 cleavage in HT-29, 769-P, WM35, WM 164, Hela, SBc-L2, WM9, and THP-1 cell lines.

Mazandarani et al. [75] reported the antioxidant and anti-inflammatory potential of acetone extract of *O*. *dichroanthum* because of existing secondary metabolites (phenol, flavonoid, and anthocyanin) in it. Badruddeen et al. [76] investigated the hydroalcoholic fraction of *O*. *bracteatum* having protective activity towards the stress generated by immune imbalance functions in Sprague Dawley rats and concluded that it can be used for the curing of numerous immune deficiency disorders. The chloroform extract of *O*. *khyberianun* exhibited antibacterial and antifungal effects [77]. Mašković et al. [78] reported that aqueous extract from *O*. *aucheriana* showed antioxidant activity evaluated by different *in vitro* assays viz. phosphomolybdenum, free radical scavenging, and lipid peroxidation assays. In addition, aqueous extract of *O*. *aucheriana* showed a cytotoxic effect against human rhabdomyosarcoma (RD) and human cervix carcinoma (Hep2c) with half maximal inhibitory concentration (IC_50_, concentration needed to kill cells by 50% as compared to untreated control) values of 40.34 μg/mL and 50.57 μg/mL, respectively due to presence of rosmarinic, gallic and p-hydroxybenzoic acids. A study reported by Engel et al. [79] represents that extract of *O*. *hispida* showed satisfactory cytotoxic outcomes on MG-63, Saos-2 (bone) and BT-20, and MCF-7 (breast) cancer cell lines. Albaqami et al. [80] also conclude the cytotoxic potential of *O*. *bracteatum* towards BT549 (breast), PC-3 (human prostate), and A549 (lung) cancer cells in a concentration-dependent manner via upregulating the activity of caspase-3. The petroleum ether and an aqueous fraction of *O*. *hispidum* Wall. showed antiproliferative and antidiabetic effects against HepG2 (hepatocellular carcinoma) cell line [81]. Safavi et al. [66] found that cyclohexane extract of *O*. *dichroantha* stimulates migration of Hs27 (normal human skin fibroblasts) cells and promotes angiogenesis. Furthermore, the analysis of LC-MS/MS showed the presence of β,β-dimethylacrylalkannin, shikonin, and β,β-dimethylacrylshikonin (arnebin-1). These phytochemicals are responsible for wound healing effects by modulating the process of inflammation, angiogenesis, and tissue regeneration. Çalhan and Gündoğan [82] proposed that the extract of *O*. *sericeum* exhibited cytotoxicity against MCF-7 (human breast cancer) cells in a concentration-dependent manner. Tlili et al. [83] described that methanolic extract from *O*. *polyantha* and *O*. *mollis* showed remarkable antioxidant properties due to the abundance of numerous phytochemicals. Demir et al. [84] reported that *O*. *armeniacum* root extract also showed cytotoxicity towards WiDr (colon), A549 (lung), and HepG2 (human liver) cancer cell lines because of existing phenolic compounds in it. The hydroalcoholic extract of *O*. *bracteatum* Wall. exhibited an antiproliferative effect against rat peritoneal mast cells [28]. Ethyl acetate fraction of *O*. *bracteata* showed apoptosis-inducing and antimigration potential by enhancing the expression level of tumor suppressor protein 53 (p53) and decreasing the level of B-cell lymphoma-2 (BCL-2), cyclin E, cyclin-dependent kinase 2 (CDK2), and mortalin results induced cell death in MG-63 cells via accumulating ROS level and protein kinase B (AKT)/glycogen synthase kinase 3β (GSK3β)/cyclin E pathway [85]. In addition, ethanolic extract (Obeth) of *O*. *bracteata* modulated the expression level of p53, COX-2, and nuclear factor kappa B (NF-κB) in carbon tetrachloride (CCl_4_)-treated male Wistar rats and showed hepatoprotective activity by decreasing the expression of p53, cyclooxygenase (COX)-2 and NF-κB in a dose-dependent manner in the liver of male Wistar rats [25]. An increase in the expression level of p53, COX-2, and NF-κB was due to enhanced oxidative stress, inflammation, and changes in cellular DNA [86]. Matrix metalloproteinases (MMPs) play a key role in metastasis and assist molecules to stimulate cancer cell invasion and metastasis in tumor cells. Several clinical reports highlighted that MMP-2 and MMP-9 levels are upregulated in many tumors [87], which is correlated with poor survival in patients with tumors [88]. As a result, MMPs, particularly MMP-2 and MMP-9, could be interesting targets for antimetastatic drugs. Cao et al. [89] reported that shikonofuran E isolated from *O*. *paniculatum* showed anti-inflammatory effects in murine macrophage (RAW 264.7) cells via modulating expression levels of the extracellular signal-regulated kinase (ERK), p38, NF-κB inhibitor alpha (IκBα), nuclear factor kappa light chain enhancer of activated B cells (NF-κB), inducible nitric oxide synthase (iNOS), COX-2, and mitogen-activated protein kinase (MAPK).

**Figure 3.**
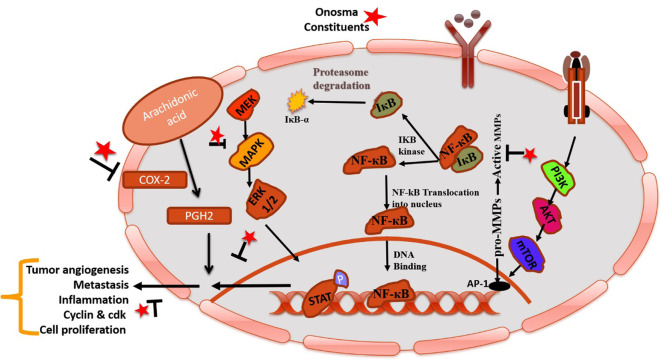
Pictorial representation of mechanistic insight of phytoconstituents of *O*. in cancer by modulating COX-2 and NF-κB. IκB-α: NF-κB inhibitor alpha; Iκβ: I-kappa-beta; IKB: inhibitor of NF-κB kinase; STAT: signal transducer and activator of transcription; MEK: mitogen-activated protein kinase kinase; PGH2: prostaglandin H2; mTOR: mammalian target of rapamycin; PI3K: phosphatidylinositol-3-kinases; AP-1: activator protein 1; P: phosphorylation; T mark: blockage; arrow mark: increase metastasis/inflammation; star mark: phytoconstituents present in *O*. species

MAPK family is a key component of the intracellular signaling network that maintains homeostasis [90]. This pathway involves several signaling mechanisms and phosphorylation events that show the main role in tumorigenesis. Main cellular events, viz. cell growth, differentiation, proliferation, apoptosis, and migration are sustained by activated kinases through extracellular signals [91, 92]. Modulation of this pathway by therapeutic agents could be targeted for the treatment of cancer. In the Unani system of medicine, the plant *O*. was also used to treat stress, imbalance of body homeostasis, and instabilities of the normal physiology of the body including psychological (behavioral fluctuations), immunological and hormonal changes. Possible mechanisms of action of *O*. constituents in cancer represents in Figure 3.

According to the study by Asif et al. [93], aqueous ethanolic extract of *O*. *bracteatum* did not show any toxicity in Swiss albino mice (male) rats even at dosages of 1, 3, 5, along with 10 g/kg. Therefore, it is suggested that *O*. has numerous phytoconstituents and less toxicity and can be used to prepare many medicinal drugs. In recent years, the medicinal and industrial importance of *O*. species has attracted the interest of researchers to discover more novel phytoconstituents and biological activities [94].

## Traditional uses

The plant species of *O*. L. have traditionally been used as stimulants for kidney irritation rheumatism, bladder pain, and heart palpitations due to their astringent, diuretic, cooling, and demulcent properties [95]. In India, it is known to cure fever, nervous disorders, and hypertension [38]. Additionally, the different species of the genus *O*. are employed in the management of inflammation, pain, and illnesses conditions like tonsillitis, hemorrhoids, and bronchitis [96]. Traditional Turkish medicine uses the roots of *O*. *argentatum* Hub.-Mor. to cure burns and wounds. In the traditional medicine of the province of Lorestan, an oily extract from the roots of the plant Tashnehdary (*O*. *chlorotricum*) is applied topically to treat wounds [53].

## Industrial uses

The roots of the plant *O*. *hispidum* Wall. is said to be the source of Ratanjot, a red dye frequently used to color foods, oils, and pharmaceutical preparations [97]. It has also been used as an adulterant to food preparations and spices like chili powder because of its hue [78].

## Conclusions

Taxonomic, morphological, and karyological data of genus *O*. L. have complex and contentious patterns. Thousands of related plants were described based on their slight physical differences. These plants are either the subspecies of other recognized plants or only one or two references. Most of these plants are of the same species; however, numerous researchers use separate names/titles due to a lack of taxonomical information. This could be because of morphological differences induced by various climatic circumstances. Only about 37 plants have accurate taxonomical data, in accordance with botanical lists of the Missouri Botanical Garden, Kew, and the Royal Botanical Garden. In addition to these countries, these genus’ plants are widely dispersed in India, Switzerland, Iran, Romania, Anatolia, China, Pakistan, Syria, and Sri Lanka. This genus differs in petal morphology, such as corolla color, shape and size, exterior nutlet characteristics, and ornamentation. The presence of naturally occurring alkanins and shikonin along with other phytoconstituents like onosmone, hispidone, onosmins, and uplandicine is abundant in the genus *O*. as far as concerned phytochemicals. Moreover, the presence of flavonoids, ferulic acid, and vanillic acid is also explored, and they might be the source of analgesic, anti-inflammatory, wound-healing, and antibacterial activities. Local people use the roots of this genus for hypertension, astringent, pain, demulcent, diuretic, fever, and inflammation as well as various other medical conditions. Overall, plants possess various biological activities such as anticancer, antioxidant, anti-inflammatory, antimicrobial, anti-diabetic, antitussive, and spasmolytic. The chief goal of the present review is to create a permanent body of genus literature on plants resource information to support upcoming research and global human involvement.

In this review article, the anticancer effects of *O*. L. are explored, with a focus on its anti-inflammatory, antiproliferative, apoptotic, and antimetastatic properties. The information presented in this review might be useful for further unraveling the molecular pathways modulated by the potent phytochemicals present in the *O*. L. More pre-clinical and clinical research is required to support the concept that *O*. and its isolated bioactive constituents can be utilized successfully in cancer therapy for different types of cancer. In the future, further toxicity studies should be performed by the researchers to ensure the safe application of novel compounds present in *O*. L. Moreover, nanotechnology-based methods could also be opted to enhance targeted delivery in the cancer microenvironment.

## Abbreviations

COX-2: cyclooxygenase 2
LC: liquid chromatography
MAPK: mitogen-activated protein kinase
MMPs: matrix metalloproteinases
MS: mass spectrometry
NF-κB: nuclear factor kappa B
NMR: nuclear magnetic resonance
*O*.: *Onosma*

## Declarations

### Copyright

© The Author(s) 2022.
